# Refraction and topographic risk factors for early myopic regression after small-incision lenticule extraction surgery

**DOI:** 10.1038/s41598-024-59327-6

**Published:** 2024-04-16

**Authors:** Chia-Yi Lee, Yu-Ting Jeng, Chen-Cheng Chao, Ie-Bin Lian, Jing-Yang Huang, Shun-Fa Yang, Chao-Kai Chang

**Affiliations:** 1 Institute of Medicine, Chung Shan Medical University, Taichung, Taiwan; 2 Nobel Eye Institute, No. 13-5, Gongyuan Rd., Zhongzheng Dist., Taipei, 100008 Taiwan; 3 Department of Ophthalmology, Jen-Ai Hospital Dali Branch, Taichung, Taiwan; 4 Department of Optometry, MacKay Junior College of Medicine, Nursing, and Management, Taipei, Taiwan; 5 Institute of Statistical and Information Science, National Changhua University of Education, Changhua, Taiwan; 6 Department of Medical Research, Chung Shan Medical University Hospital, Taichung, Taiwan; 7 Department of Optometry, Da-Yeh University, Chunghua, Taiwan

**Keywords:** Small incision lenticule extraction, Myopic regression, Corneal curvature, Central corneal thickness, Spherical equivalent, Risk factors, Eye diseases

## Abstract

We sought to evaluate the topographic risk factors for early myopic regression after small-incision lenticule extraction (SMILE). A retrospective case‒control study was conducted, and individuals who underwent SMILE surgery were enrolled. Among them, 406 and 14 eyes were categorized into the nonregression and regression groups, respectively. The preoperative and postoperative parameters in the two groups were collected, including spherical refraction (SE), axial length (AXL) and topographic data. A generalized linear model was adopted to analyze the difference in each parameter between the two groups. After 6 months, UCVA decreased in the regression group, and SE increased in the regression group (both P < 0.05). The increase in the CCT at the thinnest point (P = 0.044), flat corneal curvature (P = 0.012) and TCRP (P = 0.001) were significantly greater in the regression group. Regarding the risk factors for myopic regression, preoperative SE, preoperative sphere power, preoperative AXL, preoperative flat corneal curvature, preoperative SA, early postoperative SE, early postoperative sphere power, early postoperative AXL and early postoperative CCT difference were significantly greater in the regression group (all P < 0.05). The SE, sphere power, AXL, preoperative flat corneal curvature, preoperative SA, and postoperative CCT difference correlate with early myopic regression after SMILE.

## Introduction

Keratorefractive surgeries have been applied for the correction of refractive errors for decades, and many keratorefractive surgeries are currently available^1^. Keratorefractives can be grossly divided into three types: photorefractive keratectomy, laser in situ keratomileusis (LASIK), and small-incision lenticule extraction (SMILE)^2^. Recently, the number of patients receiving SMILE refractive surgery has gradually increased, possibly because of the small incision and high comfort^3^. The efficiency and predictability of LASIK and SMILE are not markedly different^4,5^, but the postoperative corneal sensitivity was better for SMILE surgery in the early literature^6^. In contrast, the optical density, which may lead to blurry vision, was greater during the early postoperative intervals in patients who underwent the SMILE procedure than in those who underwent the LASIK procedure^7^.

Although the safety of keratorefractive surgeries is acceptable according to previous studies, certain postoperative complications can still develop in patients who undergo LASIK or SMILE^8^. The major postoperative complications of keratorefractive surgeries include dry eye disease, corneal abrasion, epithelial ingrowth, diffuse lamellar keratitis, infectious keratitis and corneal ectasia^9,10^. In addition, myopic regression is both a natural course and an uncommon postoperative complication of LASIK and SMILE surgeries^11–13^. Natural myopic regression years after refractive surgery does not cause significant visual disturbance^14,15^, while early myopic regression can contribute to decreased vision^16^. In previous studies, approximately 4 and 20% of SMILE and LASIK patients with early myopic progression, respectively, underwent an enhancement procedure to recover vision^10,17^. Accordingly, identifying risk factors for early myopic regression after refractive surgery cannot be overemphasized.

The risk factors for early myopic regression in the LASIK procedure have been reported^11,18^. In a previous study, high myopia severity, a large optic zone, and female sex were correlated with a greater incidence of early myopic regression after LASIK^11,18^. Nevertheless, few studies have investigated the possible risk factors for early myopic regression in individuals who undergo the SMILE procedure. Furthermore, myopia progression in the general population can result from axial elongation and steepening/thickening of the cornea^19^, but the exact mechanism of early myopic regression in the SMILE procedure has not been fully elucidated.

Consequently, the purpose of the current study was to evaluate the clinical manifestations and risk factors for early myopic regression in individuals who undergo the SMILE procedure. The axial length and topographic features were analyzed in the current study.

## Materials and methods

### Ethics declaration

The current study adhered to the Declaration of Helsinki in 1964 and its later amendments. In addition, the current study was approved by the Institutional Review Board of National Changhua University of Education (project code: NCUEREC-110-081). Written informed consent was waived by the above institution.

### Participant selection

A retrospective case‒control study was conducted at the Nobel Eye Clinic, Kaohsiung Branch, which is a clinic specializing in refractive surgery that is located in southern Taiwan. First, we collected data from all individuals who underwent SMILE surgery at the Kaohsiung branch of the Nobel Eye Clinic from 2021–2022. The individuals enrolled in the current study, or the patients who underwent SMILE surgery in our clinic, met the following criteria: (1) aged between 18 and 55 years, (2) had preoperative myopia for at least − 1.00 diopters (D), and (3) had a preoperative refractive error within the correction range of SMILE surgery, which means that the sphere power was lower than − 10.00 D and the cylinder power was lower than − 5.00 D. On the other hand, the following exclusion criteria were used to exclude individuals with prominent ocular morbidity that could interfere with the outcome of SMILE surgery: (1) uncorrected visual acuity (UCVA) lower than 20/400 on the Snellen chart; (2) the existence of cataracts; (3) the development of clinically significant subclinical keratoconus or other corneal ectasic diseases; (4) severe dry eye disease, active recurrent corneal erosion, central corneal opacity, and corneal neovascularization; (5) the presence of prominent retinal diseases such as proliferative diabetic retinopathy, vitreous hemorrhage, retinal detachment and macular pucker; (6) the development of clinically significant glaucoma; (7) the presence of preceding eyeball rupture and severe ptosis that cover the pupil; (8) the presence of optic nerve atrophy, active optic neuritis or ischemic optic neuropathy; (9) unstable refractive status with a refractive error progression of more than 0.5 D in the previous two years; (10) pregnancy status; and (11) active systemic inflammatory diseases such as diabetes mellitus, systemic lupus erythematous, Sjogren’s syndrome, thyroid disease, rheumatic arthritis, ankylosing spondylitis, and systemic sclerosis. After the selection procedure, 420 eyes from 210 individuals who underwent SMILE surgery were enrolled in the present study. Then, we checked the postoperative refraction status of our patients, and patients who experienced myopic regression for more than − 0.75 D within 6 months after SMILE surgery were regarded as having early myopic regression. Finally, a total of 14 eyes and 406 eyes were categorized into regression and nonregression groups, respectively.

### Surgical technique

All the SMILE procedures in the current study were performed by two experienced refractive specialists (Y.-T.J. and C.-K.C.). The SMILE procedure was completed by one femtosecond laser device (Visuamax 500, Carl Zeiss, Göschwitzer Str., Jena, Germany). The optic zone was set as 5.5–6.9 mm based on the ablation depth and pupil size, and the corneal incision was set as 3.0 mm and built at 105 degrees. After the angle kappa was checked by microscopy with the assistance of corneal topography and the coaxial sighted corneal light reflex, the cornea was fixated by a suction ring. After the emission of the femtosecond laser, a special spatula was applied to dissect the upper and lower interfaces of the lenticule, and the lenticule was then extracted by forceps. After surgery, levofloxacin eye drops and prednisolone eye drops were applied for approximately one week, followed by replacement with sulfamethoxazole and fluorometholone eye drops for another three weeks.

### Ophthalmic examination

All the patients who received SMILE procedures underwent the same preoperative/postoperative ophthalmic examinations in our clinic performed by two experienced optometrists who have practiced for more than 10 years. The preoperative examinations included procedures and measurements as follows: manifest refraction with best corrected visual acuity (BCVA), cyclopegic refraction of both sphere and cylinder power via an autorefractor (KR-8900, Topcon, Itabashi-ku, Tokyo, Japan), central corneal thickness (CCT) of both the corneal apex and thinnest part, steep and flat corneal curvature, corneal cylinder power, total higher order aberration (HOA) at 4 mm pupil size, spherical aberration (SA) at 6 mm pupil size, corneal diameter (CD), and total corneal refractive power (TCRP) by a tomographic machine (Oculus Pentacam, OCULUS Optikgeräte GmbH, Münchholzhäuser, Wetzlar, Germany), and axial length (AXL) by a biometry machine (IOL Master 500, Carl Zeiss, Göschwitzer Str., Jena, Germany). Postoperative examinations included UCVA, BCVA, manifest sphere power and cylinder power, CCT, corneal curvature, corneal cylinder power and the AXL. Postoperative examinations via devices identical to those used for preoperative examinations were also performed. In addition, the optic zone, side-cut depth, cap thickness, residual stromal thickness (RST) and lenticule thickness during SMILE surgery were recorded. The data before the surgery, one week after the surgery, one month after the surgery, three months after the surgery and 6 months after the surgery were obtained. Notably, the spherical equivalent was regarded as the sphere power plus half of the cylinder power in the current study, and the difference in CCT was regarded as the CCT of the apex minus the CCT of the thinnest area in the current study.

### Statistical analysis

SPSS version 20.0 (SPSS Inc., Chicago, Illinois, USA) was used for all the statistical analyses in the present study. The Shapiro–Wilk test was employed to check the normal distribution of regression and nonregression groups, which revealed a non-normal distribution. Besides, the statistical power was 0.93 under 0.05 alpha value and the medium effect size with the usage of G ∗ power version 3.1.9.2 (Heinrich-Heine-Universit¨at, D¨usseldorf, Germany). Descriptive analysis was used to evaluate age, sex, refraction status, AXL, surgical parameters and topographic characteristics, and the Mann–Whitney *U* test and Fisher’s exact test were subsequently used to compare these indices between the two groups. The Mann–Whitney *U* test was also applied to evaluate the differences in efficiency and predictability between the two groups six months after SMILE surgery. Regarding the changes in UCVA and refractive status as SE during the study period, a generalized linear model was applied to compare the differences between the regression and nonregression groups with adjustments for age, sex, preoperative refractive status, preoperative AXL, preoperative topographic characteristics, and surgical parameters. Then, the adjusted odds ratios (aORs) with 95% confidence intervals (CIs) of UCVA and manifest refraction between the two groups were calculated. In addition, the generalized linear model was also used to analyze (1) the changes in AXL and topographic features from one week to six months postoperatively, (2) the differences in preoperative AXL and topographic features, and (3) the differences in AXL and topographic features one week (i.e. early postoperative period) after SMILE surgery between the two groups. The aORs and 95% CIs of the above three comparisons were then calculated after adjusting for age, sex, and preoperative cycloplegic refraction. A P value < 0.05 was defined as statistically significant, and a P value lower than 0.001 was defined as P < 0.001 in the present study.

## Results

The baseline characteristics of the regression and nonregression groups are presented in Table 1. Age and sex were similar between the two groups, and BCVA did not significantly differ between the two groups (all P > 0.05). The regression group exhibited significantly greater SE and sphere and cylinder powers than did the nonregression group for both the manifest refraction and cycloplegic refraction (all P > 0.05). Regarding the AXL and topographic parameters, the AXL was significantly longer in the regression group than in the nonregression group (P = 0.027), while all the topographic parameters demonstrated similar values between the two groups (all P > 0.05) except for the SA (P = 0.022). The lenticule thickness was significantly greater in the regression group (P = 0.006), while the other surgical indices were similar between the two groups (all P > 0.05).

**Table 1:** Basic characteristics of the study population.

Character	Nonregression group, (N = 406)	Regression group, (N = 14)	P value
Age	31.36 ± 5.12	33.86 ± 5.65	0.075
Sex (male:female)	176:243	9:5	0.104
BCVA	1.00 ± 0.04	1.00 ± 0.01	0.855
Manifest refraction			
SE	− 5.69 ± 2.07	− 7.26 ± 2.69	0.006*
Sphere	− 5.20 ± 2.02	− 6.45 ± 2.88	0.025*
Cylinder	− 0.98 ± 0.73	− 1.63 ± 2.11	0.002*
Cycloplegic refraction			
SE	− 5.35 ± 2.10	− 6.94 ± 3.00	0.007*
Sphere	− 4.82 ± 2.04	− 6.10 ± 3.16	0.024*
Cylinder	− 1.05 ± 0.79	− 1.66 ± 1.44	0.007*
AXL	25.96 ± 1.04	26.60 ± 1.61	0.027*
CCT			
Apex	547.71 ± 30.97	563.36 ± 27.71	0.063
Thinnest	543.26 ± 31.08	559.42 ± 27.20	0.055
Difference	4.45 ± 2.39	3.93 ± 1.82	0.420
Steep corneal curvature	44.16 ± 1.34	45.60 ± 1.28	0.123
Flat corneal curvature	42.76 ± 1.28	43.93 ± 0.68	0.063
Corneal cylinder	− 1.41 ± 0.73	− 1.67 ± 1.33	0.199
Total HOA	0.17 ± 0.05	0.17 ± 0.07	0.794
SA	0.20 ± 0.09	0.27 ± 0.12	0.022*
CD	12.21 ± 0.27	12.18 ± 0.36	0.557
TCRP	42.17 ± 1.25	42.43 ± 0.66	0.083
Optic zone	6.46 ± 0.39	6.34 ± 0.31	0.220
Side-cut depth	13.03 ± 3.79	12.14 ± 2.57	0.385
Cap thickness	107.49 ± 9.05	107.14 ± 9.94	0.887
RST	315.50 ± 31.69	311.50 ± 30.59	0.642
Lenticule thickness	120.11 ± 26.79	140.29 ± 36.45	0.006*

Fisher’s exact test was used for comparisons of sex ratios between groups.

Mann–Whitney *U* test was used for comparisons of age, BCVA, refraction and topographic/surgical parameters between the two groups.

*AXL* axial length, *BCVA* best-corrected visual acuity, *CCT* central corneal thickness, *CD* corneal diameter, *HOA* higher order aberration, *N* number, *RST* residual stromal thickness, *SA* spherical aberration, *SE* spherical equivalent, *TCRP* total corneal refractive power.

*Denotes a significant difference between groups.

**Figure 1:**
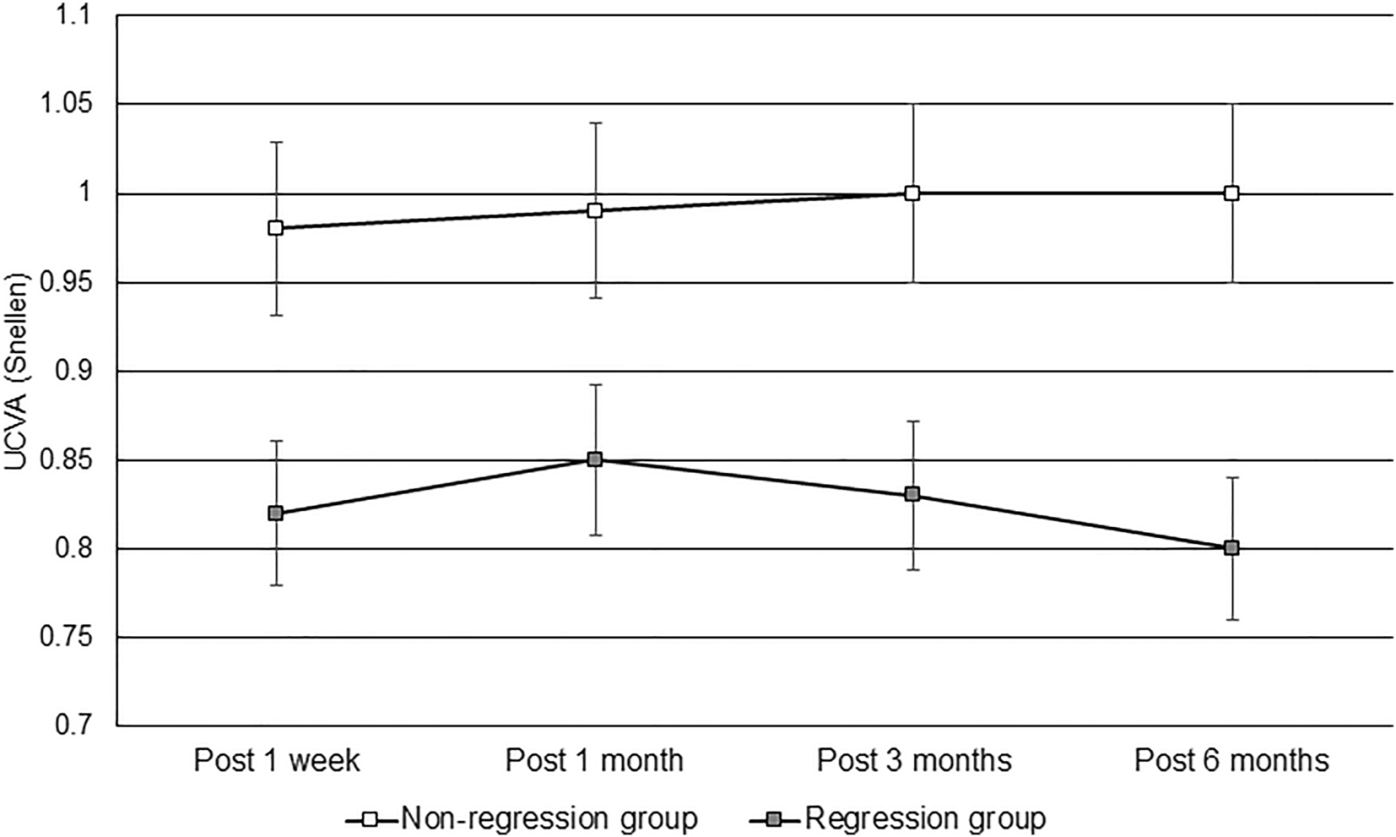
Changes in uncorrected visual acuity between the nonregression and regression populations. *UCVA* uncorrected visual acuity. Generalized linear model was adopted for comparison of UCVA between groups with adjustments for age, sex, preoperative refractive status, preoperative AXL, preoperative topographic characteristics, and surgical parameters.

**Figure 2:**
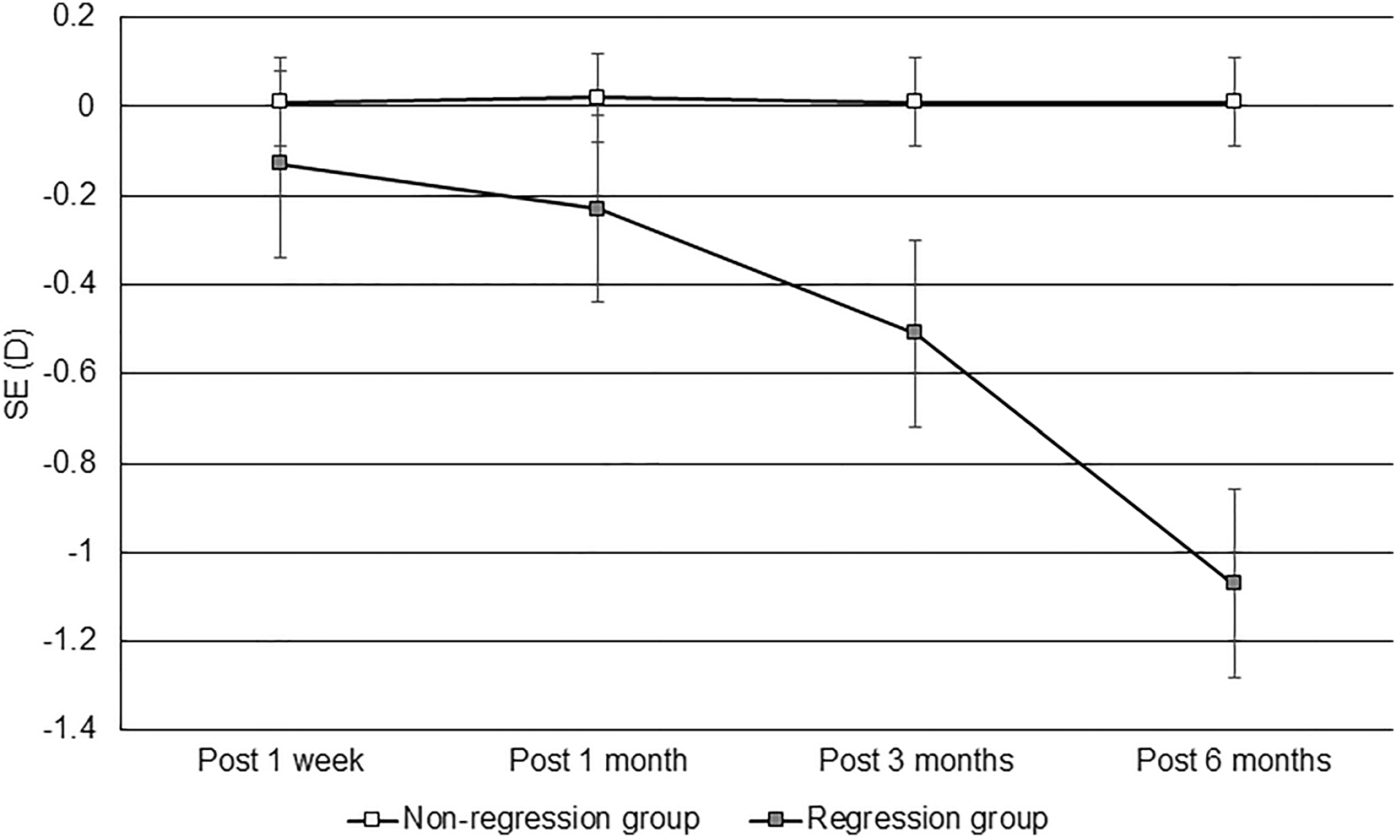
Changes in the spherical equivalent between the nonregression and regression populations. *D* diopter, *SE* spherical equivalent. Generalized linear model was adopted for comparison of SE between groups with adjustments for age, sex, preoperative refractive status, preoperative AXL, preoperative topographic characteristics, and surgical parameters.

After 6 months, the UCVA in the nonregression group was 0.99 ± 0.02, which was significantly better than the 0.80 ± 0.16 in the regression group (P < 0.001), and the BCVA showed a marginal difference between the nonregression and regression groups (1.00 ± 0.07 versus 0.96 ± 0.09, P = 0.053). The SE and sphere and cylinder powers of the manifest refraction were significantly lower in the nonregression group than in the regression group (all P < 0.001). AXL was significantly longer in the regression group than in the nonregression group (P = 0.029), while the topographic parameters were not significantly different between the two groups (all P > 0.05) (Table 2). The trend of UCVA was better in the regression group than in the nonregression group (aOR: 1.556, 95% CI 1.231–2.014, P = 0.007) (Fig. 1), and the SE trended toward myopic shift in the regression group compared with the nonregression group (aOR: 2.342, 95% CI 1.827–3.646, P < 0.001) (Fig. 2).

**Table 2:** Comparison of the efficacy and predictability indices between the two groups 6 months after surgery.

Index	Nonregression group, (N = 406)	Regression group, (N = 14)	P value
UCVA	0.99 ± 0.02	0.80 ± 0.16	< 0.001*
BCVA	1.00 ± 0.07	0.96 ± 0.09	0.053
Manifest refraction			
SE	0.01 ± 0.12	− 1.07 ± 0.75	< 0.001*
Sphere	0.02 ± 0.15	− 0.81 ± 0.85	< 0.001*
Cylinder	− 0.01 ± 0.10	− 0.48 ± 0.45	< 0.001*
AXL	25.84 ± 1.04	26.47 ± 1.60	0.029*
CCT			
Apex	449.64 ± 30.29	446.93 ± 28.88	0.741
Thinnest	447.08 ± 30.34	444.43 ± 28.72	0.748
Difference	2.57 ± 1.67	2.50 ± 1.16	0.958
Steep corneal curvature	38.94 ± 1.86	39.39 ± 2.32	0.084
Flat corneal curvature	38.05 ± 1.86	38.43 ± 2.01	0.071
Corneal cylinder	− 0.89 ± 0.43	− 0.97 ± 0.71	0.536
TCRP	35.42 ± 2.15	35.01 ± 1.18	0.276

Mann–Whitney *U* test was used for comparisons of postoperative visual acuity, refraction and topographic parameters between groups.

*AXL* axial length, *BCVA* best-corrected visual acuity, *CCT* central corneal thickness, *N* number, *SE* spherical equivalent, *TCRP* total corneal refractive power, *UCVA* uncorrected visual acuity.

*Denotes a significant difference between groups.

Concerning the AXL and topographic changes during the follow-up interval, the increase in the CCT at the thinnest point (P = 0.044), flat corneal curvature (P = 0.012), and TCRP (P = 0.001) were significantly greater in the regression group than in the nonregression group, while other topographic parameter changes were similar between the two groups (all P > 0.05) (Table 3). Regarding the risk factors for myopic regression, both the preoperative and early postoperative SE, sphere power, and AXL were significantly greater in the regression group (all P < 0.05). Additionally, the preoperative flat corneal curvature, preoperative SA, and early postoperative CCT difference were significantly greater in the regression group than in the nonregression group (all P < 0.05) (Table 4).

**Table 3:** Postoperative axial and topographic changes between the two groups.

Parameter	Nonregression group, (N = 406)	Regression group, (N = 14)	P value
AXL	− 0.03 ± 0.19	− 0.03 ± 0.04	0.961
CCT			
Apex	5.27 ± 7.96	5.64 ± 6.83	0.863
Thinnest	5.41 ± 7.80	6.36 ± 6.11	0.044*
Difference	− 0.14 ± 1.41	− 0.71 ± 1.59	0.039*
Steep corneal curvature	0.12 ± 0.67	0.26 ± 0.45	0.116
Flat corneal curvature	0.05 ± 0.76	0.25 ± 0.54	0.012*
Corneal cylinder	− 0.04 ± 0.38	0.07 ± 0.19	0.267
TCRP	0.06 ± 0.14	0.18 ± 0.11	0.001*

Generalized linear model was adopted for comparisons of axial and topographic changes between groups after adjusting for age, sex, and preoperative cycloplegic refractions.

*AXL* axial length, *BCVA* best-corrected visual acuity, *CCT* central corneal thickness, *N* number, *SE* spherical equivalent, *TCRP* total corneal refractive power, *UCVA* uncorrected visual acuity.

*Denotes a significant difference between groups.

**Table 4:** Correlations between myopic regression and preoperative and early postoperative factors.

Factor	aOR	95% CI	P value
Preoperative			
Manifest refraction- SE	1.465	1.002–1.847	0.002*
Manifest refraction- sphere	1.720	1.342–2.145	< 0.001*
Manifest refraction- cylinder	1.032	0.795–1.435	0.524
AXL	1.678	1.445–2.018	0.001*
CCT-Apex	1.321	0.693–1.572	0.357
CCT-thinnest	1.011	0.715–1.214	0.554
CCT-difference	0.923	0.884–1.084	0.065
Steep corneal curvature	1.324	0.773–1.820	0.090
Flat corneal curvature	1.665	1.401–2.232	0.001*
Corneal cylinder	1.083	0.802–1.592	0.447
Total HOA	1.257	0.945–1.863	0.089
SA	1.654	1.147–2.395	0.001*
CD	1.196	0.869–1.416	0.095
TCRP	1.575	0.988–1.934	0.064
Early postoperative			
Manifest refraction- SE	1.859	1.553–2.647	< 0.001*
Manifest refraction- sphere	2.374	1.667–3.005	< 0.001*
Manifest refraction- cylinder	0.924	0.521–1.604	0.615
AXL	1.527	1.382–1.996	0.003*
CCT-apex	1.242	0.926–1.875	0.099
CCT-thinnest	1.339	0.870–1.716	0.165
CCT-difference	1.648	1.008–2.275	0.027*
Steep corneal curvature	1.108	0.692–1.741	0.343
Flat corneal curvature	1.529	0.904–1.862	0.076
Corneal cylinder	1.111	0.856–1.406	0.413

Generalized linear model was adopted for comparison of preoperative and early postoperative factors (i.e. refraction, axial length and topographic parameters) between groups after adjusting for age, sex, and preoperative cycloplegic refractions.

*aOR* adjusted odds ratio, *AXL* axial length, *CCT* central corneal thickness, *CD* corneal diameter, *CI* confidence interval, *HOA* higher order aberration, *N* number, *SA* spherical aberration, *SE* spherical equivalent, *TCRP* total corneal refractive power, *UCVA* uncorrected visual acuity.

*Denotes a significant difference between groups.

## Discussion

In the present study, the changes in the thinnest CCT, flat corneal curvature and TCRP were greater in patients who experienced early myopic regression. Moreover, high preoperative and early postoperative SE, sphere strength, and AXL correlated with a greater risk of myopic regression. In addition, the high preoperative flat corneal curvature, high preoperative SA and high early postoperative in the CCT difference were also associated with myopic regression after SMILE surgery.

There are several hypotheses for early myopic regression after refractive surgery^18^. Thickening of the corneal epithelium is one of the possible mechanisms of myopic regression after LASIK surgery according to a previous study^16^. In a previous study, the CCT increased 3 months after the LASIK procedure in patients with myopic regression^20^. The effect of laser ablation on the cornea may trigger a wound healing process that causes proliferation of the corneal epithelium^21^. In addition, postoperative dry eye disease after LASIK can damage the ocular surface and alter the structure of the corneal epithelium^22^. In previous research, the corneal epithelium was shown to proliferate in the presence of high levels of inflammatory cytokines such as growth factor and insulin-like growth factor-1^23,24^, which also exist in the cornea after the LASIK procedure^25^. Another possible mechanism of early myopic regression in refractive surgery is the forward shift of corneal curvature^16^. The tensile strength of the cornea after refractive surgery, including photorefractive keratectomy, LASIK and SMILE, decreases, and SMILE is associated with the highest residual corneal stiffness^26^. A weakened corneal structure could contribute to anterior movement of the cornea and subsequent development of myopia^27,28^. This hypothesis was supported by the fact that TCRP after refractive surgery increases, although dominant myopic regression was not observed^29^. Nevertheless, myopic development can result from both axial elongation and corneal curvature steepening^19^, and whether postoperative axial elongation occurs in patients who experience early myopic regression has not been investigated.

In the present study, the AXL levels in the regression and nonregression groups were nearly identical; thus, the possibility of AXL elongation-related myopic regression may be low. On the other hand, the thickness of the thinnest part of the cornea and the difference between the CCTs in the corneal apex and the thinnest cornea increased significantly in the regression group in the present study, and the flat corneal curvature increased significantly in the regression group. Moreover, the TCRP increased more significantly in the regression group than in the nonregression group, which agreed with the increased TCRP after refractive surgery^29^, but we further highlighted that the change in the TCRP was more pronounced in those with myopic regression. The above results in the current study may further prove that myopic regression after SMILE surgery results from changes in corneal structures, similar to the mechanism of myopic regression after LASIK surgery.

The risk factors for early myopic regression included increased preoperative and early postoperative sphere power and AXL. In previous studies discussing the risk factors for myopic regression after LASIK, the degree of myopia, greater postoperative central corneal thickness, aspherical ablation and large transitional zone of laser ablation were correlated with a greater risk of myopic regression^11,18^. Another study also demonstrated the association between the pre-subjective sphere and the risk of myopic regression in the LASIK and SMILE procedures^12^. However, few studies have evaluated ocular factors for early myopic regression after SMILE surgery. A previous study evaluated the risk factors for the early astigmatic regression of SMILE surgery^30^, while the current study surveyed the potential risk factors for the early myopic regression of SMILE surgery despite both studies were conducted in Taiwan. Also, the current study recorded the change of visual acuity and refraction continuously while the previous study only collected postoperative data for once^30^, and certain information like AXL, SA, CD and TCRP were absent in the previous literature^30^. To our knowledge, this may be preliminary research on axial and topographic factors for early myopic regression in individuals who undergo SMILE surgery with adequate follow-up frequency and preoperative information. Higher SE and sphere power are correlated with a greater degree of myopia, and high myopia is a known risk factor for myopic regression after LASIK surgery^16^. In addition, patients with high myopia usually have a long AXL, and the presence of a long AXL was correlated with early myopic regression after LASIK surgery in a previous study^31^. Consequently, SMILE and LASIK share similar risk factor (i.e., high myopia status) for early myopic regression.

In addition, the high preoperative flat corneal curvature, high preoperative SA and high early postoperative CCT difference between the apical and thinnest corneas were also associated with myopic regression after SMILE surgery. In a previous study evaluating corneal curvature and myopic regression after the LASIK procedure, the increase in corneal curvature was also related to myopic regression^11^. We speculate that a greater flat corneal curvature indicates a greater baseline corneal curvature that may be prone to extension after SMILE surgery. Additionally, a study including more than 1000 participants revealed a correlation between preoperative SA and myopic regression in SMILE patients^12^, and the current study corresponds to this publication. HOA and CD were significantly associated with myopic regression in that study^12^, contrary to the results of the present study. Nevertheless, the aORs of total HOA and CD in the current study presented a marginally positive correlation with myopic regression, and these findings may become statistically significant if more participants are included. Due to the difference in the CCT and its relationship to myopic regression, corneal epithelial remodeling in patients with myopic regression leads to different degrees of epithelial thickening in different corneal areas^32^. As a consequence, the presence of a high CCT difference may indicate such a phenomenon, but further research is warranted to prove this concept.

Concerning the changes in UCVA and refraction after SMILE surgery during the 6-month period, the regression group exhibited significantly worse visual and refractive outcomes than did the nonregression group. The UCVA in the nonregression group improved gradually after SMILE surgery, while the UCVA in the regression group decreased one month after surgery. In addition, the sphere power in the regression group continued to increase. The changes in UCVA and refraction during the postoperative period between the two groups were still significantly different after adjusting for age, sex and initial refraction status, indicating that myopic regression after SMILE surgery is multifactorial as we discussed in earlier paragraphs^16,18^. On the contrary, the cylinder power also showed a significant difference between the regression and nonregression groups 6 months postoperatively. However, the average cylinder power at 6 months in the regression group after SMILE surgery was approximately − 0.48 D, which is a common degree of astigmatism in the general population^33^. Moreover, the degree of reduction in astigmatism after surgery was similar between the two groups. Thus, myopic regression may not be necessarily correlated with astigmatic changes, and the etiology of astigmatic regression needs further evaluation.

Regarding the efficiency and predictability of SMILE surgery in the current study, the average UCVA in the whole study group one month postoperatively was 0.95 on the Snellen chart, and 88% of individuals reached a UCVA of 20/25 or better. In a previous publication, the percentage of patients with a UCVA greater than 20/25 or greater was 100% after 2 years^34^. Another study reported that 82% and 96% of patients achieved a UCVA better than 20/25 three months after SMILE surgery^35^. The efficiency results of the current study are compatible with those of previous studies^34,35^.

For refraction, the SE one month postoperatively for the whole study population was -0.01 D in the current study. Previous studies demonstrated postoperative SEs ranging from − 0.28 to − 0.38 D^35,36^, similar to the findings of our study. Moreover, excluding those patients with myopic regression, all the patients in the nonregression group had an SE lower than − 0.50 D at 6 months after SMILE surgery. Accordingly, the predictability of the SMILE in our institution could be guaranteed. The survival rate of myopic regression in the current study was 96.67% after 6 months, which is also not inferior to that reported in previous studies^12^.

There are some limitations in the current study. First, the retrospective design of the current study may have diminished the homogeneity of the study group. In addition, the numbers of patients in the nonregression and regression groups were highly imbalanced; the number of patients in the nonregression group was approximately 40-fold greater than the number of patients in the regression group, possibly due to the relatively small number of patients with early myopic regression in clinical practice. However, such an imbalance of patient numbers can lead to statistical bias even with the application of a generalized linear model. In addition, some corneal parameters, such as the tomographic biomechanical index, corneal hysteresis, corneal resistance factor, root-mean-square, anterior chamber depth and specific HOAs, such as coma and trefoil, were not evaluated in the current study, and the above factors may also influence changes in the configuration of the cornea and possible myopia formation after SMILE surgery. Finally, SMILE surgery was performed by two surgeons; thus, differences in the surgical techniques used by the two surgeons may exist. Nevertheless, the two surgeons who performed the SMILE surgery in the current study were skilled and used a similar protocol for SMILE surgery. As a consequence, the influence of different surgeons on the outcome of SMILE surgery might be minimal.

In conclusion, early myopic regression after SMILE surgery results from topographic changes, including changes in CCT, TCRP and flat corneal curvature. Furthermore, a high preoperative refraction, long AXL, high flat corneal curvature, high preoperative SA and large difference of CCT are risk factors for early myopic regression. Consequently, repeated topographic exams and surgical plan modifications may be considered for such populations. Further large-scale prospective studies evaluating the topographic risk factors for early astigmatic regression are needed.

## Data Availability

The data used in the current study are available from the corresponding author upon reasonable request.

## Abbreviations

LASIK: Laser in situ keratomileusis
SMILE: Small incision lenticule extraction
D: Diopter
UCVA: Uncorrected visual acuity
CCT: Central corneal thickness
K: Corneal curvature
AXL: Axial length
HOA: Higher order aberration
SA: Spherical aberration
CD: Corneal diameter
TCRP: Total corneal refractive power
aOR: Adjusted odds ratio
CI: 95% Confidence interval
N: Number
SD: Standard deviation
